# New Clinicoepidemiologic Profile of Cutaneous Leishmaniasis, Morocco

**DOI:** 10.3201/eid1309.070946

**Published:** 2007-09

**Authors:** Mohamed Rhajaoui, Abedelmajeed Nasereddin, Hajiba Fellah, Kifaya Azmi, Fatima Amarir, Amer Al-Jawabreh, Suheir Ereqat, Joseph Planer, Ziad Abdeen

**Affiliations:** * Institut National d’Hygiène, Rabat, Morocco; † Al-Quds University, East Jerusalem, Palestine; ‡ Manchester College, North Manchester, Indiana, USA; 1 These authors contributed equally to this article.

**Keywords:** cutaneous leishmaniasis, polymerase chain reaction, Leishmania tropica, Morocco, dispatch

## Abstract

During the past 20 years, cutaneous leishmaniasis has emerged as a major public health threat in Morocco. We describe distribution of *Leishmania major* and *L. tropica* in Morocco and a new focus of cutaneous leishmaniasis due to *L. infantum.* We recommend using molecular techniques to diagnose suspected leishmaniasis cases.

Leishmaniasis, a vectorborne parasitic disease, affects 1.5–2 million people annually. In >100 countries whose populations are at risk for the disease, the disease inflicts a high economic cost (*1*,*2*). Additionally, large-scale emergence and reemergence have been recently reported in many Mediterranean countries, including Morocco (*1*,*3*).

Cutaneous leishmaniasis (CL) caused by *Leishmania major* has been reported in Morocco since 1914 (*4*); until recently, however, it was largely confined to arid Saharan regions (*4,**5*). In 2001, the Moroccan Ministry of Health (MMH) reported 2,028 CL cases caused by *L. major* and *L. tropica* (*6*). Of the 3 clinically important *Leishmania* species (*L. major, L. tropica, L. infantum*), *L. tropica* has the largest geographic distribution and is considered a public health threat by the MMH. *L. tropica* CL has been reported in Azilal, Essaouira, Taza, Fes, the province of Chichaoua, and central Morocco (*5*,*7*–*10*).

Accurate diagnosis and treatment of CL requires positive identification of the causative species of parasite (*11*). Often, however, traditional diagnostic methods such as analysis of clinical symptoms, microscopic identification, and parasite culture are performed in place of molecular diagnostic techniques, such as PCR. Problematically, all *Leishmania* species have similar morphology, and several species capable of causing both CL and visceral leishmaniasis (VL) may exist in the same locales.

We update the current epidemiologic profile of *Leishmania* spp. in Morocco by using archived clinical samples tested by PCR. We provide economic and epidemiologic rationales for our recommendation that species-specific identification be performed for all cases of suspected leishmaniasis.

## The Study

**Figure 1:**
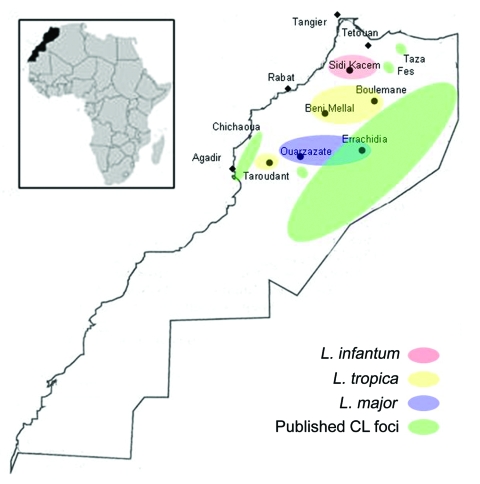
Molecular epidemiology of cutaneous leishmaniasis in Morocco. *L., Leishmania*.

Tissue samples were taken from 27 patients with suspected CL who had consulted the health centers from March 2005 to March 2006. Local reference laboratories evaluated all stained slides by light microscopy and positively identified *Leishmania* amastigotes. Patients had no history of travel and were assumed to been infected in Morocco; all received free intralesional injections of meglumine antimoniate (Glucantime; Sanofi-Aventis, Bridgewater, NJ, USA) until total recovery, according to the protocol in the MMH leishmaniasis control manual. Samples were collected in areas of Morocco known for high CL incidence: north (Sidi Kacem), center (Beni Mellal and Boulemane), southeast (Errachidia), and southwest (Taroudant and Ouarzazate) (Figure 1).

DNA extraction and PCR analysis by amplification of the ribosomal internal transcribed spacer 1 (ITS1), using stained slides, was performed as described by Schonian et al. (*12*). We used 0.6-nM primers and PCR-Ready Supreme mix (Syntezza Bioscience, Jerusalem, Israel) in 25 μL of total reaction. *Leishmania* DNA (10 ng/reaction) from reference strains *L. tropica* (MHOM/AZ/1974/SAF-K27), *L. major* (MHOM/TM/1973/5ASKH), and *L. infantum* (MHOM/TN/1980/IPT1) were used as positive controls. Negative controls for extracted DNA and PCR analysis were included. After amplification, the PCR product was digested with 1.5 μL *BsuR*I endonuclease (MBI Fermentas, Burlington, Ontario, Canada), and all digested products were analyzed by agarose gel electrophoresis (*12*).

All patients had classic symptoms of CL, from small erythematous papules to nodules and ulcerative lesions. Patients’ ages varied from 1.25 to 70 years. The sample comprised 44% male and 56% female patients (Table). Papular lesions, nodular lesions, or both were present in 30% of the CL patients; ulcerative lesions, in 52%. Neither the papular/nodular nor the ulcerative forms correlated with a particular *Leishmania* species. The erythematous clinical form was present in 18% of total case-patients and in 63% of case-patients from the Sidi Kacem region.

**Table:** Distribution of cutaneous leishmaniasis, 27 patients, Morocco, 2005–2006

Geographic origin	No. cases	Age range, y	Sex	Clinical lesions	*Leishmania* species
Taroudant	2	5–20	1M, 1F	Nodular (2)	*L. tropica*
Beni Mellal	4	3–11	3M, 1F	Ulcerative (4)	*L. tropica*
Boulemane	7	1.25–60	4M, 3F	Ulcerative (5) Papulonodular (2)	*L. tropica*
Ouarzazate	3	0.25–52	1M, 2F	Ulcerative (2) Nodular (1)	*L. major*
Errachidia	3	3–39	1M, 2F	Ulcerative (3)	*L. major*
Sidi Kacem	8	2–70	2M, 6F	Erythematous (5) Papulonodular (2) Nodular (1)	*L. infantum*

**Figure 2:**
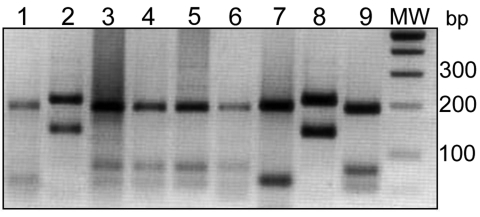
Comparison between endonuclease *BsuR*I digestion patterns of internal transcribed spacer 1–PCR products from clinical samples and reference *Leishmania* strains. Clinical samples, lanes 1–6: lane 1, Boulmane; lane 2, Ouarzazate; lanes 3–6, Sidi Kacem. Reference strains, lanes 7–9: lane 7, *L. tropica* (MHOM/SU/1974/SAF-K27); lane 8, *L. major* (MHOM/TM/1973/5ASKH); lane 9, *L. infantum* (MHOM/TN/1980/IPT1). MW, DNA molecular weight marker in base pairs (bp).

Undigested ITS1 amplicons from the 27 slides produced a band of 300–350 bp (data not shown), which confirmed the presence of *Leishmania* DNA. Band patterns from the digested samples were compared with digested standards for each reference strain and identified the parasite species (Figure 2) as follows: *L. major,* 3 samples each from Ouarzazate and Errachidia; *L. tropica,* 2 samples from Taroudant, 4 from Beni Mellal, and 7 from Boulemane; *L. infantum,* 8 samples from Sidi Kacem.

## Conclusions

CL caused by *L. major* or *L. tropica* and VL caused by *L. infantum* have been reported in Morocco (*5*–*9*). PCR on archived tissue samples enabled us to investigate the epidemiology of CL in disease-endemic regions of Morocco and identify those species responsible for this disease in several new foci (Table). Our results, together with those of previous studies (*5*–*9*; unpub. data from MMH, 2001), indicate that CL caused by *L. tropica* is found throughout the center of the country in a band stretching from the Atlantic Ocean along the length of the Atlas Mountains almost to the Mediterranean Sea. CL caused by *L. major* is present in the desert region south of the Atlas Mountains in a strip bordering the Sahara Desert (Figure 1).

We report on a focus of CL in Morocco caused by *L. infantum*, 8 samples from Sidi Kacem. In Morocco, the only previous human CL case caused by *L. infantum* was reported in 1996, within an active focus of VL (*13*). The northern coastal regions of Morocco are endemic for human and canine VL. As in other VL-endemic regions surrounding the Mediterranean Sea, this disease is caused by *L. infantum* (*3*). Although it is unusual for this parasite to cause CL, our finding is similar to a recent report from Tunisia, where *L. infantum* was shown to cause sporadic CL in regions endemic for VL. It appeared to have emerged in a new region of the country and was suggested to be more prevalent than originally indicated (*14*).

CL and VL overlap in many provinces of central Morocco; anthroponotic foci of *L. tropica* CL are found in Fes and Taza (*7*–*9*) (Figure 1), not far from existing VL foci including Sidi Kacem. Furthermore, several cases of canine VL caused by *L. tropica* have been reported in regions where canine VL is caused by *L. infantum*.

The nodular form of CL was caused by all 3 species; ulcerative lesions were seen only with CL caused by *L. tropica* and *L. major*. Of the 8 patients in Sidi Kacem with *L. infantum* infection, 5 showed the atypical erythematous papular form. These findings agree with results of studies in northern Morocco (*7*). The overlapping distribution of parasite species, causing diseases with similar clinical pictures, demonstrates the need for additional epidemiologic and ecologic studies of CL in conjunction with species identification. This is especially important as traditional methods of determining infection from patient history and microscopic examination prove increasingly unreliable. PCR can be performed rapidly on fresh or archived samples and does not require culturing of large amounts of parasites. In addition, PCR costs have come down considerably, and costs can be further reduced by sending samples by regular mail to a central facility.

Recent studies document the emergence of new *Leishmania* foci and the coexistence of multiple *Leishmania* species in the same geographic locale, including much of northern Africa (*14*). We recommend that treatment protocols, particularly in areas of coexistence, be predicated on diagnosis of not only the clinical form—CL versus VL—but additionally the disease-causing species.

In Morocco, local physicians and healthcare administrators often do not realize that different species of *Leishmania* require differential treatments, which can result in a failure to diagnose more serious disease. Risk for metastatic lesions with *L. major* is almost zero. However, recurrent failure of local treatments (paromomycin and intralesional sodium stibogluconate) against *L. tropica* was evident (*11*). Because *L. tropica*, and now *L. infantum,* cause both VL and CL, a physician treating a cutaneous lesion may overlook visceral disease, and a host of costlier health problems may ensue. A simple, sensitive PCR test could easily reduce such risk. Further surveillance of cases and suspected cases from these foci should confirm the results of this limited study.

Recent implementation of PCR-based diagnosis in an outbreak in northern Algeria increased the positive diagnosis of CL by 69% over cases diagnosed by using microscopy alone (*15*). Furthermore, anthroponotic CL caused by *L. tropica* is limited to parts of southern Europe, Asia, and Africa; diagnosis and treatment of the disease at its earliest stage is of paramount importance for reduction of the human reservoir. Failure to promptly diagnose and treat all cases will result in continued dissemination of the parasite.
